# Effects of Extremely Low-Frequency Electromagnetic Fields on Neurogenesis and Cognitive Behavior in an Experimental Model of Hippocampal Injury

**DOI:** 10.1155/2017/9194261

**Published:** 2017-11-12

**Authors:** Mohammad Hassan Sakhaie, Mansoureh Soleimani, Bagher Pourheydar, Zahra Majd, Pezhman Atefimanesh, Sara Soleimani Asl, Mehdi Mehdizadeh

**Affiliations:** ^1^Cellular and Molecular Research Center and Department of Anatomy, Iran University of Medical Sciences, Tehran, Iran; ^2^Department of Anatomy, Arak University of Medical Sciences, Arak, Iran; ^3^Urmia University of Medical Sciences, Faculty of Medicine, Neurophysiology Research Center, Department of Anatomy, Urmia, Iran; ^4^Oncopathology Research Center, Iran University of Medical Sciences, Tehran, Iran; ^5^Cellular and Molecular Research Center, Faculty of Advanced Technologies in Medicine, Iran University of Medical Sciences, Tehran, Iran; ^6^Endometrium and Endometriosis Research Center, Hamadan University of Medical Sciences, Hamadan, Iran; ^7^Anatomy Department, School of Medicine, Hamadan University of Medical Sciences, Hamadan, Iran; ^8^Cellular and Molecular Research Center, Faculty of Advanced Technologies in Medicine, Department of Anatomy, Iran University of Medical Sciences, Tehran, Iran

## Abstract

Exposure to extremely low-frequency electromagnetic fields may induce constant modulation in neuronal plasticity. In recent years, tremendous efforts have been made to design a suitable strategy for enhancing adult neurogenesis, which seems to be deterred due to brain senescence and several neurodegenerative diseases. In this study, we evaluated the effects of ELF-EMF on neurogenesis and memory, following treatment with trimethyltin chloride (TMT) as a neurotoxicant. The mice in all groups (*n* = 56) were injected with BrdU during the experiment for seven consecutive days to label newborn cells. Spatial memory was assessed by the Morris water maze (MWM) test. By the end of the experiment, neurogenesis and neuronal differentiation were assessed in the hippocampus, using immunohistochemistry and Western blot analysis. Based on the findings, exposure to ELF-EMF enhanced spatial learning and memory in the MWM test. ELF-EMF exposure significantly enhanced the number of BrdU+ and NeuN+ cells in the dentate gyrus of adult mice (*P* < 0.001 and *P* < 0.05, resp.). Western blot analysis revealed significant upregulation of NeuroD2 in ELF-EMF-exposed mice compared to the TMT-treated group (*P* < 0.05). These findings suggest that ELF-EMF might have clinical implications for the improvement of neurodegenerative processes and could help develop a novel therapeutic approach in regenerative medicine.

## 1. Introduction

Extremely low-frequency electromagnetic fields (ELF-EMFs) are emitted from home appliances and medical devices. Frequencies between 0 and 300 Hz are conventionally called extremely low frequencies (ELF) [1] Numerous studies have reported the influence of magnetic fields on biological systems. ELF-EMF exerts certain effects on brain activity, function of the nervous system, and cognitive behaviors [2]. Recently, ELF-EMF has been proposed for the modulation of hippocampal functions, including cell proliferation, neurogenesis, and behavioral activities [3, 4]. A study by Cuccurazzu et al. showed that ELF-EMF exposure (1 mT; 50 Hz) can be an effective strategy for increasing *in vivo* hippocampal neurogenesis [3]. Moreover, ELF-EMF leads to an increase in synaptic plasticity in the perforant pathway of the dentate gyrus (DG) [5]. In addition, it can suppress neuronal apoptosis and promote cell survival in the nervous system [6]. According to a study by Perez et al., nonthermal repetitive EMF shocks induce an antiaging hormetic effect and reduce cell death in T lymphocytes and fibroblast cell lines during lethal stress [7]. Transcranial magnetic stimulation enhances neurogenesis in the subventricular zone of the brain and prevents motor alterations in rats induced by the nigrostriatal lesion [8]. Hippocampal proliferation facilitates synaptic connections, which modulates the consolidation of long-term spatial memory [9]. In a study by Podda et al., exposure to ELF-EMF (1 mT; 50 Hz; 3.5 h/day for 6 days) improved learning and performance on memory tasks, which is correlated with enhanced neurogenesis [4]. Moreover, in a previous study, chronic exposure to ELF-EMF in a 50 Hz magnetic field (2 mT) for one or four hours exerted positive effects on the acquisition and maintenance of spatial memory [10]. Several factors might contribute to the promotional effect of chronic exposure on learning and long-term memory. ELF-EMF is deemed to stimulate the upregulated expression of proteins in voltage-gated Ca^2+^ channels and increases Ca^2+^ influx [11]. It is well established that a postsynaptic rise in intracellular Ca^2+^ concentration plays a major role in long-term potentiation (LTP) induction of CA1 pyramidal neurons as one of the involved cellular mechanisms in learning and memory [10]. In this study, we used trimethyltin chloride (TMT) to propose a model of hippocampal injury for examining neurogenesis, neuronal maturation, and neurobehavioral effects of ELF-EMF in adult male BALB/c mice. TMT, an organotin compound with neurotoxicant effects in the limbic system and hippocampus, is considered a useful tool to obtain an experimental model of neurodegeneration [12]. TMT is regarded as a neurotoxicant substance, which can be useful for studying response to injury with respect to the pattern of degeneration and neuronal apoptosis in the hippocampal DG of mice and rats. TMT significantly elevates the level of cytokines including tumor necrosis factor (TNF)-alpha, TNF-beta, and interleukin-1 alpha. On the other hand, TMT does not affect the level of glucocorticoids, and, consequently, it cannot downregulate the injury response in rat hippocampus, following systemic injection [13]. According to a study by Halladay et al., TMT induces perturbation of the polysialylated neural cell adhesion molecule in the hippocampus and brain cortex; also, TMT exposure can result in spatial learning impairments [14].

Based on the aforementioned background, the aim of this study was to determine the effects of ELF-EMF on cell proliferation and behavioral cognition in response to TMT neurotoxicity in the hippocampal DG of BALB/c mice.

## 2. Materials and Methods

### 2.1. Animals

In total, 56 adult male BALB/c mice (6-7 weeks old; Pasteur Institute of Iran) were maintained in an animal house under controlled conditions (temperature: 21 ± 2°C, relative humidity: 45 ± 5%, and a 12 h light/12 h dark cycle) with access to food and water ad libitum. All animal procedures were performed according to the Guidelines for the Care and Use of Experimental Animals and were approved by the Veterinary Ethics Committee of Iran University of Medical Sciences. The sequence of specific primers used in this study is shown in Table 1.

### 2.2. EMF Generation System

ELF-EMF was generated by two solenoids connected to an alternating-current power generator. Each solenoid was made of 380-round turn coil (19 cm in diameter and 17.5 in length) and a long magnetic wire twisted around a Plexiglass cylinder. ELF-EMF-exposed animals were placed in a plastic cage inside the center of a cylinder surrounded by solenoids.

**Figure 1:**
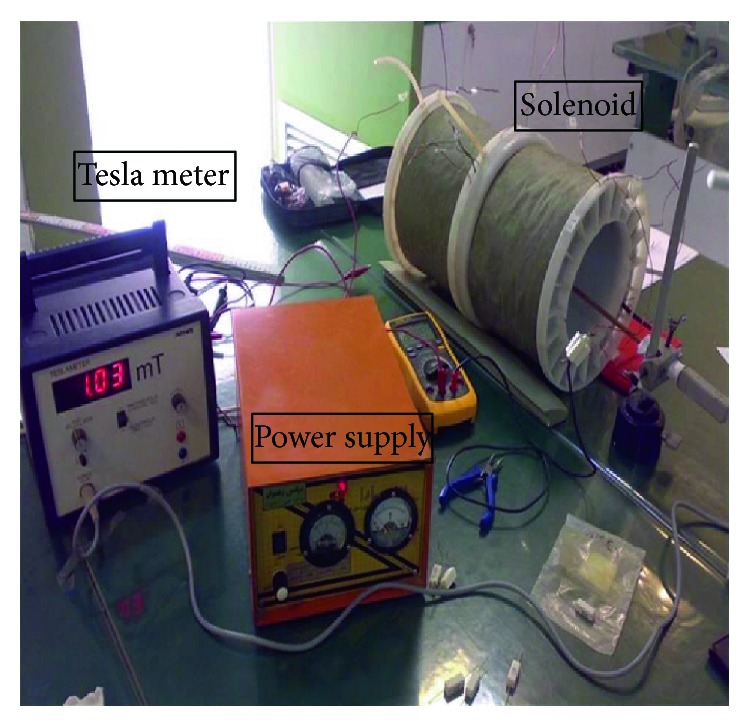
The electromagnetic exposure system. Homogeneity was defined by measuring the magnetic field intensity at different time intervals. Digital temperature sensors were placed inside the chambers. The animals were placed in a plastic cage and the whole body was exposed to electromagnetic waves.

The strength of the magnetic field was monitored with a probe connected to a Telemeter (Compensation 51662; Germany) to measure the precise intensity of EMF (Figure 1). We evaluated the effects of ELF-EMF on neurogenesis and memory, following treatment with TMT as a neurotoxicant. The temperature was regulated with an external temperature sensor to reduce the drift of the thermometer to minimum. Another similar sample holder was placed in an EMF-protected container for the control group. The animal exposure continued for six days (six hours daily).

### 2.3. Experimental Groups

**Figure 2:**
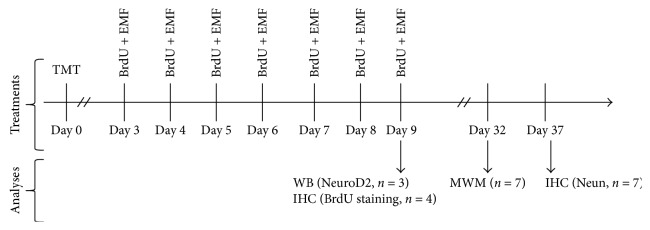
Timeline of BrdU injection + ELF-EMF exposure protocol showing the number of animals used for each group of experiments. WB: Western blot; IHC: immunohistochemistry; MVM: Morris water maze.

The animals were randomly divided into four groups (14 mice per group): (1) control group without ELF-EMF stimulation; (2) sham group receiving an intraperitoneal injection of saline without ELF-EMF stimulation; (3) TMT group receiving a single dose of TMT via intraperitoneal injection (2.5 mg/kg; Merck, Germany) without ELF-EMF stimulation; and (4) TMT + ELF-EMF group exposed to ELF-EMF (50 Hz, 1 millitesla) after TMT injection (Figure 2). The animal exposure continued for six days (six hours daily), following 72 hours after TMT injection. Since the initiation of exposure, all the groups received intraperitoneal injections (50 mg/kg) of bromodeoxyuridine (BrdU) (B9285; Sigma-Aldrich, USA).

### 2.4. Morris Water Maze (MWM) Test

Thirty-two days following the final exposure, we randomly selected seven mice to facilitate behavioral analysis in the MWM test. This test is based on the spontaneous tendency of rodents to escape water using a submerged platform in a circular tank. In our laboratory, MWM test consisted of a stainless steel tank (110 cm in diameter, 60 cm in depth) filled with water (23 ± 1°C). Several visual cues were present around the room and remained unchanged during the experiment. The maze was divided into four quadrants: northwest, northeast, southwest, and southeast. Also, the starting positions, that is, north, south, east, and west, were located around the perimeter of the tank.

A hidden circular platform (12 cm in diameter) was located at the center of the southwest quadrant and submerged 1 cm below the surface of the water. The position of the escape platform remained constant for all the animals in all training sessions. Each mouse received four training sessions per day for four consecutive days. On day five, the probe test was performed by removing the platform and allowing each mouse to swim freely for 60 sec.

An infrared video camera (Nikon, Melville, NY, USA) was mounted directly above the water maze tank to record the length of swim path (traveled distance), the time spent to reach the submerged platform (escape latency), and percentage of time spent in the target quadrant for each mouse.

### 2.5. Immunohistochemistry

Twenty-four hours after the final exposure to ELF-EMF, four mice from each group were anesthetized with ketamine (100 mg/kg) and xylazine (10 mg/kg). The mice were transcardially perfused with 4% paraformaldehyde in phosphate buffer and then sacrificed. The animal brains were removed and postfixed overnight. Then, the brains were dehydrated in the ascending alcohol series, rinsed using xylene, and infiltrated with paraffin.

Afterwards, the blocks were divided into 5 *μ*m coronal sections (postbregma region: −1.34–2.54 mm). Assessment of BrdU incorporation in the hippocampal DG region was performed as previously described [15]. Briefly, the sections were incubated in 50% formamide and 2x standard sodium citrate buffer at 65°C for 2 h and then incubated twice in 100 mM of sodium borate (pH = 8.5). Then, DNA was denatured by incubating the sections in 2 N HCl at 37°C, rinsed in phosphate-buffered saline (PBS), and blocked with 0.4% Triton X-100 in PBS and goat serum (10%) for 30 min.

The sections were incubated overnight with mouse monoclonal anti-BrdU (1 : 70; Sigma, USA) at 4°C. The sections were then incubated with a mouse monoclonal secondary antibody (HRP) (1 : 200; Abcam, Cambridge, UK) for 1 h and placed in a wet dark box at room temperature. Peroxidase 3,3′-diaminobenzidine (DAB) was used to visualize and induce the antigen-antibody reaction.

Finally, the sections were washed in PBS and counterstained with hematoxylin. For each animal, the average BrdU+ cell count was measured by counting five coronal sections of granular and subgranular DG layers using a light microscope with ×40 objective lens (Olympus AX70 Provis, Japan), attached to a digital camera (Olympus DP11, Japan).

We performed NeuN immunohistochemical analysis 37 days after the final ELF-EMF exposure in all the groups to assess neuronal maturation. The animal brains (*n* = 7) were sectioned into 5 *μ*m coronal sections (postbregma region: −1.34 mm to −2.54 mm). Briefly, after paraffin removal by immersion in decreasing grades of ethanol and washing with Tris-buffered saline (TBS; pH = 7.4), endogenous peroxidase was quenched with 0.3% H_2_O_2_ for 30 min. Then, the sections were exposed to autoclave antigen retrieval and incubated in a blocking solution (serum protein-free solution, Dako, Denmark).

The sections were incubated with mouse monoclonal primary antibody against the neuronal nuclear antigen (NeuN, 1 : 100; Millipore Chemicon International, MAB377) and secondary HRP-conjugated anti-mouse IgG antibody (1 : 200; Abcam, Cambridge, UK). After washing with TBS, peroxidase DAB (Dako, Denmark) was used to visualize the antigen-antibody reaction. The slides were counterstained with hematoxylin and mounted. An unbiased stereological method was used for counting NeuN+ neurons, as previously described [16].

### 2.6. Western Blot Analysis

Following deep anesthesia, the animals (three mice per group) were sacrificed by decapitation 24 hours after the final exposure to ELF-EMF. The fresh hippocampi were rapidly dissected, frozen in liquid nitrogen, and stored at −80°C until further use. Then, the tissues were homogenized in ice-cold lysis RIPA buffer consisting of 1 : 20 RIPA buffer and a protease inhibitor cocktail.

The lysates (12,000 g) were centrifuged for 30 min at 4°C, and a 5 *μ*l aliquot of the supernatant was used to determine protein concentration. Prior to electrophoresis, the samples were denatured at 95°C for 5 min. The total protein content (100 *μ*g) was separated on sodium dodecyl sulfate (SDS) polyacrylamide gel. Then, it was transferred to a nitrocellulose membrane in a semidry transfer tank at 80 volts for 50 min with a transfer buffer containing Tris-base, 192 mM of glycine, 0.1% SDS, and 20% methanol.

The membranes were blocked with 5% skim milk in TBS, containing 0.5% Tween 20, and were incubated with primary antibodies directed against mouse monoclonal anti-NeuroD2 antibody (1 : 1500; Abcam, Cambridge, UK) and glyceraldehyde 3-phosphate dehydrogenase (GAPDH, 1 : 1000; Abcam, Cambridge, UK) for 2 h.

After washing the membranes in a mixture of TBS and Tween 20 three times, they were reincubated with secondary alkaline-phosphatase-conjugated antibody (1 : 5000; Abcam, Cambridge, UK) for 1 h. Finally, the bands were detected with a chromogenic substrate (5-bromo-4-chloro-3-indolyl phosphate) in the presence of nitro blue tetrazolium. Densitometric measurements of proteins were analyzed by UVIdoc software (Houston, USA).

### 2.7. Statistical Analysis

Statistical analysis was performed using SPSS version 16. Data were presented as mean ± SEM. One-way analysis of variance (ANOVA) and Tukey's tests were used to analyze the differences between the groups. *P* value less than 0.05 was considered statistically significant.

## 3. Results

### 3.1. Effects of ELF-EMF on Learning and Spatial Memory

**Figure 3:**
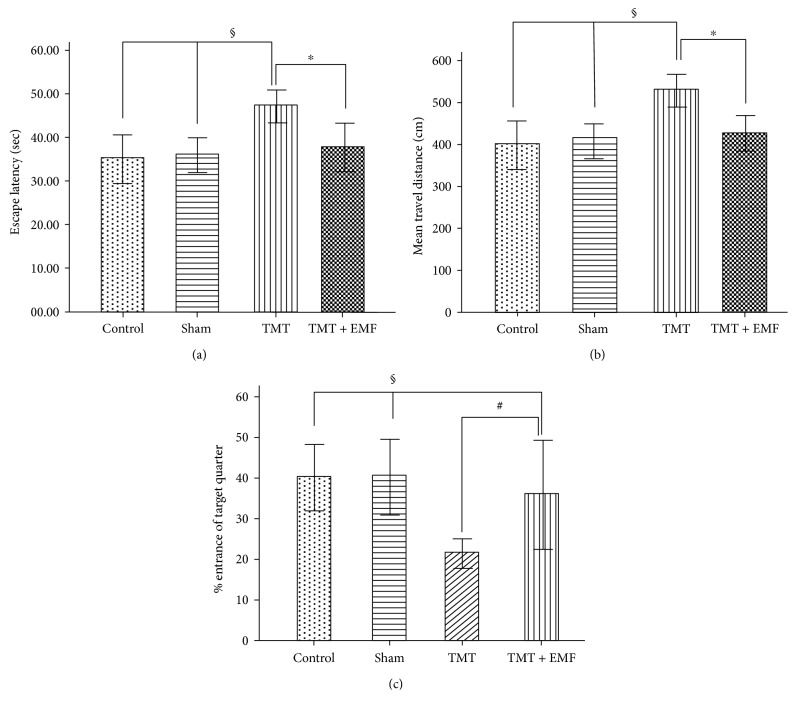
Morris water maze (MWM) test. (a) The mean latency to find the hidden platform, (b) traveled distance, and (c) percentage of the entrance to the target quadrant. Data are presented as mean ± SEM (^∗^*P* < 0.05, ^§^*P* < 0.01, and ^#^*P* < 0.001).

Analysis during four days of training showed that the TMT-treated group spent more time to find the hidden platform (escape latency) than the other groups (Figure 3(a)). Longer escape latency is indicative of more severe spatial memory deficits. Also, according to post hoc test results, the TMT group was significantly different from the control and sham groups (*P* < 0.01).

According to the results, ELF-EMF caused a significant decline in escape latency compared to TMT treatment (*P* < 0.05). As shown in Figure 3(b), a significant difference in the traveled distance was observed between TMT-treated and control groups (*P* < 001). Also, the traveled distance was shorter in TMT-treated mice receiving ELF-EMF compared to the TMT-treated group (*P* < 0.05).

Moreover, in this study, the percentage of entrance to the target quadrant in the probe trial session was investigated (Figure 3(c)). The results showed that the control and sham groups spent more time in the target quadrant compared to the TMT-treated group (*P* < 0.001). We also observed a significant difference between TMT-treated and TMT + ELF-EMF groups (*P* < 0.01).

### 3.2. Effects of ELF-EMF Exposure on Neurogenesis in the Hippocampal DG of Mice

**Figure 4:**
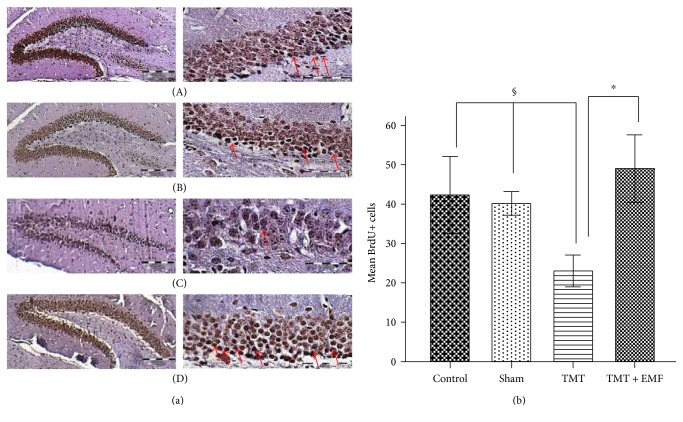
(a) Micrograph of BrdU-stained coronal sections in the dentate gyrus (DG) of the hippocampus: (A) control group, (B) sham group, (C) TMT-treated group, and (D) TMT + ELF-EMF group (scale bar: 200 *μ*m in the right panel and 500 *μ*m in the left panel). (b) The graph shows the mean number of BrdU+ cells (^∗^*P* < 0.001, ^§^*P* < 0.01).

In order to evaluate the effects of ELF-EMF on neurogenesis in hippocampal DG of mice, we performed BrdU immunostaining. BrdU incorporation revealed that TMT decreased the number of proliferated cells compared to the control and sham groups (*P* < 0.01; Figures 4(a) and 4(b)). On the other hand, exposure to ELF-EMF significantly increased the number of BrdU+ neurons due to cell proliferation compared to the TMT-treated group (*P* < 0.001), (df = 3; *F* = 13.45; *P* value = 0.000).

### 3.3. Effects of ELF-EMF Exposure on NeuN+ Neurons

**Figure 5:**
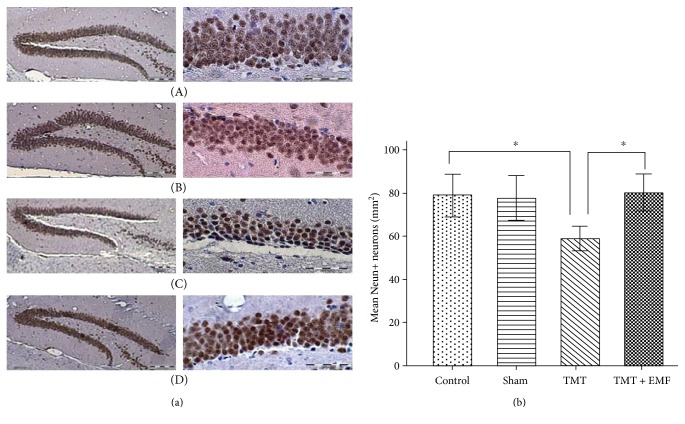
Photomicrograph of coronal sections in the dentate gyrus (DG) of the hippocampus. (a) Density of anti-NeuN marker in the study groups: (A) control group, (B) sham group, (C) TMT-treated group, and (D) TMT + ELF-EMF group (scale bar: 200 *μ*m in the right panel and 500 *μ*m in the left panel). (b) The graph illustrates the mean number of Neun+ neurons (^∗^*P* < 0.05).

As presented in Figure 5, TMT treatment caused a decline in the number of mature neurons (NeuN+) in the DG region compared to the control group (*P* < 0.05). On the other hand, ELF-EMF exposure significantly increased the number of NeuN+ neurons compared to TMT treatment (*P* < 0.05), (df = 3; *F* = 6.92; *P* value = 0.001).

### 3.4. Effects of ELF-EMF Exposure on the Expression of NeuroD2

**Figure 6:**
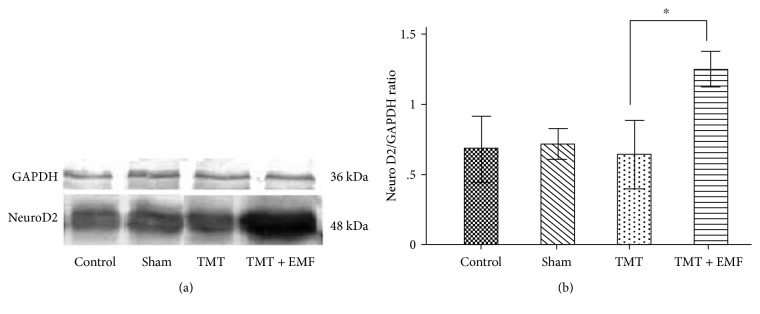
Densitometric analysis of NeuroD2 bands (at approximately 48 kDa) in hippocampal extracts of the study groups. (a and b) Representative graph of NeuroD2 expression in different groups (^∗^*P* < 0.05).

NeuroD2 plays a critical role in the promotion of neuronal survival, proliferation, and induction of neuronal differentiation. Densitometric analysis of Western blot bands showed that ELF-EMF exposure (1 mT) significantly increased the expression of NeuroD2 protein in the hippocampus compared to the TMT-treated group (*P* < 0.05; Figure 6). Western blot analysis also declared that ELF-EMF altered the expression of NeuroD2 protein in control and TMT groups that were different, but this difference was not statistically significant.

## 4. Discussion

In the present study, TMT administration caused memory impairment and reduced neurogenesis in the hippocampus. On the other hand, ELF-EMF led to an increase in the number of newly generated and mature neurons in the hippocampus. Overall, intoxication with TMT has been shown to cause cognitive and memory deficits in humans [17], as well as experimental animals [18, 19]. Some scholars believe that cognitive function is accompanied by pathological damage to the hippocampal pyramidal cells in TMT-intoxicated animals [20]. TMT leads to selective neuronal degeneration in the hippocampus through increased Bax expression and caspase-induced cell death [21]. Impairment of spatial memory and learning may be caused by a decline in D2 receptor gene expression, which possibly affects the plasticity of inhibitory circuits [19]. Another possible explanation for the inhibitory effects of TMT may be the increase in intracellular Ca^2+^ level [22]. This rise in intracellular calcium ion concentration is a result of excessive or persistent activation of glutamate-gated ion channels, which may cause neuronal degeneration [23]. ELF-EMF leads to an increase in the number of newly generated and mature neurons in the hippocampus. ELF-EMF applied at different time intervals modulates chemokine production and keratinocyte growth via inhibiting the nuclear factor kappa-light-chain-enhancer of activated B cell (NF-*κ*B) signaling pathway resulting in the possible inhibition of inflammatory processes [3]. In a previous study, Oda et al. reported that exposure to ELF-EMF (50 Hz, 300 mT) in cultured rat cerebellar neurons suppressed neuronal apoptosis and promoted survival [6] and influence intracellular proapoptotic pathways. ELF-EMF exposure could trigger alpha frequency bands in the brain, induce changes similar to neurofeedback training (of the upper-alpha frequency band), facilitate cognitive enhancement, and selectively promote the proliferation of particular neurons [1]. The present findings indicated that ELF-EMF exposure could protect the animals against the neurotoxic effects of TMT and improve learning and memory in the MWM test. In agreement with our findings, recent studies have revealed that EMF (918 MHz; 0.25 W/kg) could provide cognitive advantages to transgenic and nontransgenic mice [24] and exert positive effects on the acquisition and maintenance of spatial memory [10]. Arias et al. found that magnetic field could improve neurogenesis by altering endogenous electric fields. The stimulation consisted of an oscillatory magnetic field (60 Hz; 0.7 mT) [8]. Recently, it has been revealed that long-term exposure to high-frequency EMF could improve memory in normal mice and prevent or reverse cognitive impairment in transgenic mice with induced Alzheimer's disease [25]. According to the literature, EMF (0.16 Hz, 15 mT) exposure amplifies hippocampus-evoked potentials. Enhancement of such potentials significantly changes the efficiency of excitatory synapses leading to memory improvement [26]. In contrast with the results of the current study, some studies have shown that extended EMF exposure could cause notable long-term deficits in learning ability [27] and memory in immature mice [28]. In the mentioned studies, this difference was related to the intensity of the magnetic field (8 mT), which was higher than the intensity used in the current study (1 mT). This finding shows that magnetic field intensity plays a major role in learning and memory consolidation of immature mice. It should be noted that in these studies, the researchers used immature rodents, whereas in the current study, adult mice were used. Consistent with the present findings, Ogita et al. showed that TMT-induced damages to immature neurons could cause a decline in the number of immature neurons in the hippocampal DG [29]. Also, TMT in cultured hippocampal neurons of rats has been shown to result in an increase in intracellular free Ca^2+^ due to Ca^2+^ release from intracellular stores [30] and perturb in homeostasis, as well as necrotic and apoptotic damages to the neurons; in fact, use of TMT in culture cells caused damage to the neurons [31]. Furthermore, the results of the present study demonstrated that ELF-EMF exposure increased the number of BrdU+ cells in the hippocampal DG and enhanced cell proliferation in neurons. These results were in agreement with the findings of a previous study on mice, which indicated that ELF-EMF exposure for seven days significantly increased the number of BrdU+/DCX+ cells in the granular layer of the DG [3]. It has been suggested that exposure to ELF-EMF could induce hyperproliferation of undifferentiated precursor cells and increase neuronal differentiation of neural stem cells (NSCs) [3]. In a previous study, ELF-EMF stimulation improved the differentiation of NSCs *in vitro* by upregulating CaV1 channel expression [32]. Also, Tasset et al. reported that ELF-EMF improved neurological scores, enhanced neurotrophic factor level, and reduced neuronal loss in a rat model of Huntington's disease [33]. Cuccurazzu et al. showed that ELF-EMF increases the expression of both NeuroD1 and NeuroD2 proteins in the hippocampus and induces hyperproliferation of undifferentiated precursor cells [3]. The present findings confirmed that NeuroD2 is a proneural transcription factor, which plays a major role in neuronal commitment. In addition, ELF-EMF has been shown to increase the expression of proteins in CaV1 calcium channel and hippocampal calcium level [34], thus upregulating the expression of NeuroD proteins [35]. It has been suggested that high-frequency stimulation in awake animals elevates the brain-derived neurotrophic factor in the hippocampus, which induces neuroplasticity in the prelimbic cortex and stimulates neuropeptide Y; this neuropeptide enhances the proliferation of granule cells in the hippocampal DG [36]. Moreover, EMF has been shown to change cell membrane permeability, calcium efflux, and neuronal excitability for triggering the signal transduction cascade, which affects neuronal proliferation [37]. NeuN immunostaining is a proper biomarker for predicting delayed neuronal degeneration in the hippocampus [38]. In congruence with a study by Kurkowska et al., our findings demonstrated that TMT significantly reduces the number of NeuN immunoreactive neurons in the granular layer of DG, while ELF-EMF exposure increases the number of these neurons [39]. Overall, exposure to ELF-EMF is associated with the downregulation of Bax as a proapoptotic protein and increases the expression of antiapoptotic Bcl-2 [4]. Therefore, at least part of the beneficial effects of ELF-EMF exposure, as observed in the current study, might be attributed to intracellular signaling pathways and involve voltage-gated Ca^2+^ channels on the plasma membrane.

## 5. Conclusion

In conclusion, the present study demonstrated that ELF-EMF exposure could improve learning and memory impairment. These effects could be attributed to the induction of neurogenesis and proliferation of neurons generated from stem cells in the neurogenic layer of the hippocampus. The current findings support the initial hypothesis that this type of ELF-EMF stimulation could be used as a treatment option with promising potentials for central nervous system disorders, especially for degenerative diseases. Molecular mechanisms contributing to ELF-EMF effects might lead to the development of neuron cells and could be a promising therapeutic strategy for neurodegenerative diseases. However, further research is required to analyze the potential therapeutic effects of ELF-EMF on neurodegenerative disorders.

## Figures and Tables

**Table 1:** Sequence of specific primers used for quantitative real-time reverse transcription PCR.

Gene name		Primer sequence
COX-2	Forward	5′-GCTGTACAAGCAGTGGCAAA-3′
	Reverse	5′-CCCCAAAGATAGCATCTGGA-3′
PGE2	Forward	5′-ATCACCTTCGCCATATGCTC-3′
	Reverse	5′-GGTGGCCTAAGTATGGCAAA-3′
Caspase-3	Forward	5′-TCTACAGCACCTGGTTACTATTCC-3′
	Reverse	5′-TTCCGTTGCCACCTTCCTG-3′
MOG	Forward	5′-CAAGAAGAGGCAGCAATGGAG-3′
	Reverse	5′-CAGGAGGATCGTAGGCACAAG-3′
Bax	Forward	5′-GCAGCGGCAGTGATGGAC-3′
	Reverse	5′-TCCTGGATGAAACCCTGTAGC-3′
Bcl-2	Forward	5′-CCCTTGGCGTGTCTCTCTG-3′
	Reverse	5′-TCCTGTGATTCTCCCTTCTTCTC-3′
NG2	Forward	5′-CGTCTCTGGAAGAACAAAGGTC-3′
	Reverse	5′-AGAGTACATCATGCCGACTGC-3′
*β*-Actin	Forward	5′-GCA TCG TCA CCA ACT GGG AC-3′
	Reverse	5′-ACC TGG CCG TCA GGC AGC TC-3′

COX-2: cyclooxygenase 2, PEG2: prostaglandin E2, Caspase-3: cysteine-aspartic protease-3, MOG: myelin oligodendrocyte glycoprotein, Bax: Bcl-2-associated X protein, Bcl-2: B cell lymphoma 2, NG2: neural/glial antigen 2, *β*-actin: beta-actin.
